# Evaluation of sowing time and seed treatment fungicides on Microdochium spp. DNA quantity in winter wheat cultivars

**DOI:** 10.3389/fpls.2025.1694784

**Published:** 2025-12-02

**Authors:** Aurimas Sabeckis, Roma Semaškienė, Akvilė Jonavičienė, Karolina Lavrukaitė, Eimantas Venslovas

**Affiliations:** Department of Plant Pathology and Protection, Institute of Agriculture, Lithuanian Research Centre for Agriculture and Forestry, Akademija, Kėdainiai, Lithuania

**Keywords:** *Microdochium nivale*, seed treatment fungicides, sowing time, epidemiology, cultivar susceptibility

## Abstract

Root rots, seedling blights and snow mold diseases caused by *Microdochium nivale* and *M. majus* threaten winter wheat production in temperate regions. This study investigated the occurrence and dynamics of both pathogens in three-year field trials using quantitative PCR analysis of winter wheat cultivars sown at different times and treated with various seed treatment fungicides. Both species were consistently detected, but their prevalence varied by year: *M. nivale* was dominant under prolonged snow cover in 2023, whereas *M. majus* reached highest levels in 2022, when snow cover was less persistent. Later sowing generally reduced *M. nivale* infection in years with moderate disease pressure, although this effect was diminished under epidemic conditions. Seed treatment fungicides containing fludioxonil or succinate dehydrogenase inhibitor compounds (fluxapyroxad and sedaxane) consistently suppressed pathogen DNA levels, whereas triazole-dominant treatments showed more variable results. Cultivar-related differences were also evident, with ‘Ada’ and ‘KWS Emil’ frequently exhibiting higher infection levels than ‘Skagen’ or ‘Patras.’ These findings highlight that *Microdochium* spp. incidence is shaped by the interaction of environmental conditions, agronomic practices, and host genotype. By integrating these factors, this study advances our understanding of pathogen ecology and contributes to sustainable management strategies for winter wheat under changing climatic conditions.

## 1 Introduction

Winter wheat (*Triticum aestivum* L.) is an essential crop in temperate regions such as Northern and Eastern Europe. The vast growing scale increases the susceptibility of this vital cereal to a range of pathogenic fungi (Ristaino et al., 2021), particularly those within the *Microdochium* genus, which pose a significant threat to global food security (Gogoleva et al., 2024).

*Microdochium* species and their taxonomy have been a topic of interest for researchers for a long time. Initially, pathogens were suggested to be classified as varieties of species of the genus *Fusarium* (*Fusarium nivale* var. *nivale* and *majus*) (Wollenweber, 1930) with the later classification of *F*. *nivale* to *Microdochium nivale* by Samuels and Hallett (1983) to *Microdochium* genus. In 2005, Glynn et al. demonstrated distinct genetic heterogeneity between isolates of *Microdochium majus* and *Microdochium nivale* within a functional gene. This study provided the first sequence-based phylogenetic evidence supporting the recognition of *M. majus* and *M. nivale* as separate species (Glynn et al., 2005). Recognizing *M. nivale* and *M. majus* as distinct species is therefore important not only for taxonomy, but also for understanding their epidemiology and developing targeted management strategies in winter cereals.

Both pathogens are considered principal causative agents of pink snow mold and fusarium head blight (Tronsmo et al., 2001; Jørgensen et al., 2011; Nielsen et al., 2013), leading to considerable yield losses and grain quality reduction. They have a broad host range and can damage all winter and spring cereals affecting plants throughout basically all growth stages. *M. nivale* and *M. majus* infections can result in reduced germination, as well as pre- and post-emergence seedling death after winter, root and stem base diseases, and leaf blight (Ponomareva et al., 2021). Therefore, beyond their role in the most prominent snow mold damage, *Microdochium* spp. can contribute to significant crop losses during the early growth stages and are important causal agents of root rots and seedling blights (Jørgensen et al., 2011).

*Microdochium* spp. are soil- and seed-borne fungi that infect the plant base, particularly under conditions of prolonged soil wetness, low temperatures, and snow cover (Gorshkov et al., 2020). Infected plants often exhibit pale or necrotic leaf sheaths, reduced root systems, and poor survival during winter. Yield losses caused by *Microdochium*-related diseases can vary significantly depending on the weather conditions, cultivar susceptibility, and agronomic practices (Váňová et al., 2011).

While seed treatment fungicides are still considered as the most effective control measurement against *Microdochium* spp. it is important to use them with considered caution. Fungicide performance can vary between years and environments, therefore no single treatment consistently provides complete protection. In addition, extensive chemical control can lead to pathogen resistance for certain active ingredients, emphasizing the need for integrated strategies that combine seed treatment with appropriate sowing time and resistant cultivars (Ren et al., 2016). Active ingredients such as fludioxonil, triazoles (e.g., tebuconazole, prothioconazole, triticonazole), and SDHIs (succinate dehydrogenase inhibitors) (e.g., sedaxane, fluxapyroxad) (Walker et al., 2009) are widely applied and have shown efficacy in reducing seed and soil-borne inoculum, thereby lowering root rot and snow mold incidences (Nielsen et al., 2013; Ren et al., 2016). Although the development and use of resistant cultivars is considered the most sustainable and economical method for the control of some *Microdochium* caused diseases (Ren et al., 2016), the resistance of the most important cereal cultivars to these pathogens is currently low (Ponomareva et al., 2021). Therefore, snow mold and related diseases are an important problem in the breeding and management of winter cereals (Temirbekova et al., 2022).

Temperate climates are experiencing shifts, with longer, wetter autumns and milder winters, which create a favorable environment for *Microdochium* survival and proliferation (Juroszek and von Tiedemann, 2011). These conditions extend the period of environmental suitability and host susceptibility. These shifts could lead to increased disease pressure and altered species distributions, necessitating a deeper understanding of the factors influencing *Microdochium* dynamics in wheat protection (Gagkaeva et al., 2020). In addition, *Microdochium* pathogens gradually adapt to a warmer climate, spreading to territories with less snow and causing diverse types of plant diseases throughout the growing period (Tsers et al., 2023).

The intricate interplay between these climatic factors and agricultural practices significantly influences the incidence and severity of diseases such as pink snow mold, seedling blight, and Fusarium head blight caused by these pathogens (Gawronska and Gołębiowska-Pikania, 2016; Gagkaeva et al., 2020). Therefore, understanding the prevalence and factors influencing the incidence of *Microdochium* species is important for developing effective disease-management strategies. Although the morphological differences between *M. nivale* and *M. majus* can assist in differentiating these species in laboratory settings, molecular analysis is a more reliable tool for species identification. Notably, *M. majus* typically produces larger macroconidia that are primarily three-septate, comprising over 60% of its spores, whereas *M. nivale* tends toward fewer septa and smaller spores (Gagkaeva et al., 2020). Despite the broader average dimensions of macroconidia in *M. majus*, the conidial size ranges overlap, making morphological evaluation a useful but not fully reliable diagnostic trait (Jewell and Hsiang, 2013; Nielsen et al., 2013).

This study aimed to elucidate the impact of seed treatment fungicides, sowing time, and cultivar selection on the incidence of *Microdochium nivale* and *M. majus* in winter wheat using real-time quantitative polymerase chain reaction (qPCR) for the precise identification and quantification of fungal DNA.

## 2 Materials and methods

### 2.1 Experimental design

A three-year field study was conducted from 2021 to 2023 to investigate the occurrence of *Microdochium* spp. under various agronomic conditions. The experiments were conducted with three main factors: wheat cultivar, seed treatment, and sowing time. Four winter wheat cultivars (“Ada,” “KWS Emil,” “Skagen,” and “Patras”) were used for this experiment, each treated with four different commercial seed treatment fungicide formulations and an untreated control (**Table 1**). To evaluate the effect of sowing time, experiments were sown on two different dates: the second to third decade of September and the first decade of October, in accordance with the optimal and late sowing times of local agronomic practices. Field trials were conducted in Central Lithuania on loamy, drained soil classified as *Cambisols* with *Endocalcaric* and *Endogleyic* properties (Kochiieru et al., 2024). During the trial period winter, wheat was sown after winter oilseed rape using conventional tillage practices. Maintenance with fertilizers and herbicides was performed according to agronomical recommendations. Sowing was conducted using a Wintersteiger Kubota plot drill (Wintersteiger, Austria) in a randomized complete block design with four replicates. The seeding rate was adjusted annually to achieve a target of 450 viable seeds per square meter based on each seed lot’s germination percentage and thousand-kernel weight. Each experimental plot measured 15 m².

**Table 1:** Active ingredients and doses of seed treatment fungicides used in the field trial.

Active ingredients (g L^−1^)	Dose (L t^−1^)	Commercial name
Fludioxonil 25	1.5	Maxim 025 FS
Prothioconazole 50; Tebuconazole 10; Fludioxonil 37.5	1.0	Bariton Super
Fluxapyroxad 33.3; Triticonazole 33.3; Fludioxonil 33.3	2.0	Kinto Plus
Sedaxane 25; Fludioxonil 25; Triticonazole 20	2.0	Vibrance Star

### 2.2 Meteorological conditions

**Figure 1:**
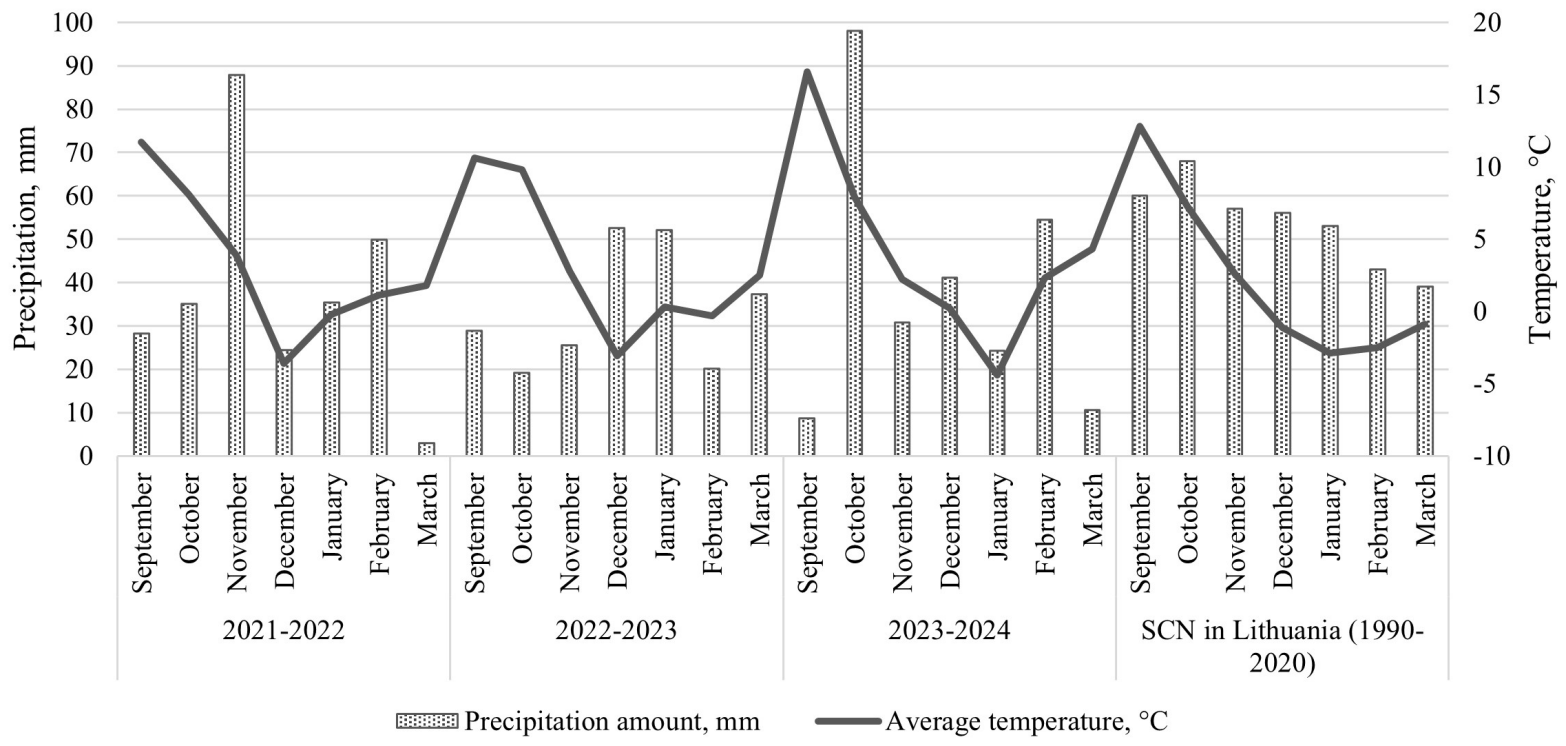
Average monthly air temperatures and total monthly precipitation in trial site, 2021–2023, compared with the SCN – Standard Climate Normal 1990–2020 (Lithuanian Hydrometeorological Service).

Meteorological data from the local Dotnuva meteorological station (**Figure 1**) revealed notable variations in autumn and winter conditions across the years of the trial. The 2021–2022 season experienced a cooler, drier autumn followed by a relatively mild winter, with a high amount of precipitation in November, mostly snowfall. In contrast, the 2022–2023 season was marked by near-average temperatures and relatively high snowfall from December to June, whereas the 2023–2024 season was characterized by fluctuating temperatures and inconsistent precipitation.

Snow cover also varied considerably; in 2022–2023, snow persisted for up to 77 consecutive days, with temperatures fluctuating around 0°C in January, creating stable, humid conditions at the soil surface. In comparison, snow cover lasted only 46 days in 2021–2022 and declined further to 36 days in 2023–2024 (Sabeckis et al., 2025).

These patterns are highly relevant to the development of *M. nivale* and *M*. *majus*, both of which are known to thrive under prolonged snow cover owing to their tolerance of low temperatures, as both species remain active under low temperature conditions. Moreover, they exhibit pathogenicity during extended cool periods, particularly when snow insulates the soil and weakens plant defenses.

### 2.3 Sampling and DNA extraction

In early spring of each year, prior to stem elongation, 30 (three plants from 10 different spots) randomly selected plants were collected from every plot. Collected winter wheat samples were cut into 1 cm long stem base segments and rinsed thoroughly under running tap water to remove soil and debris. The air-dried samples were homogenized in liquid nitrogen. A 100 mg subsample of the homogenized material was used for DNA extraction using The E.Z.N.A.^®^ SP Plant DNA Kit (Omega Biotek, Inc., United States) according to the manufacturer’s instructions.

### 2.4 Standard curves and quantitative real-time polymerase chain reaction analysis

The quantification of DNA in the sample using qPCR was based on the standard curve method. To prepare *Microdochium* standard curves, genomic DNA was extracted from pure cultures at known concentrations. Pure cultures of *M. nivale* and *M. majus* obtained from the Leibniz Institute DSMZ (Germany) were used as standards. The extracted DNA from these cultures was used to prepare standard curves. DNA concentration and purity were assessed using a biophotometer (Eppendorf, Germany). Plant standard curves were generated using DNA isolated from healthy, young leaves of the target cereal species at known concentrations, measured using a Biophotometer (Eppendorf, Germany). To construct standard curves, six 10-fold serial dilutions of DNA (1:10–1:10^6^ ) were used in the qPCR reactions (Suproniene et al., 2010; Nielsen et al., 2013; Jonavičienė, 2016).

Extracted DNA from the test samples was diluted at ratios of 1:10, 1:20, 1:50, and 1:100 to evaluate the qPCR reaction quality against the standard curves. Based on amplification performance, the dilution that produced the most consistent and reliable results was selected, and all sample DNA was subsequently diluted 1:10 with double-distilled water (ddH_2_ O) for further analysis.

Quantitative real-time PCR (qPCR) was conducted following the protocol described by Nielsen et al. (2013), with modifications from Jonavičienė (2016). Each reaction was performed in a final volume of 15 µL, comprising 7.5 µL of Power SYBR^®^ Green PCR Master Mix (Applied Biosystems, USA), 300 nM of each primer, 0.5 µg µL^−1^ of bovine serum albumin (BSA; Thermo Fisher Scientific, Lithuania), and 2.5 µL of template DNA. The species-specific primers used in this analysis are listed in **Table 2**.

**Table 2:** Species-specific primers and their sequences used for qPCR analysis.

Species detected	Primer	Sequence (5′–3′)	Source
*Microdochium nivale*	Mniv1f	TTGGCTTGCACAAACAATACTTTTT	Nielsen et al. (2013)
	Mniv1r	AGCACAACAGGCGTGGATAAG	Nielsen et al. (2013)
*Microdochium majus*	Mmajus1f	AACCCCTCCCGGGTCAG	Nielsen et al. (2013)
	Mmajus1r	GGATAAACGACACTTGAAGACAGAAAA	Nielsen et al. (2013)
*Plant EF1α* (reference)	Hor1f	TCTCTGGGTTTGAGGGTGAC	Nicolaisen et al. (2009)
	Hor2r	GGCCCTTGTACCAGTCAAGGT	Nicolaisen et al. (2009)

All qPCR reactions were conducted in duplicate using a 7900HT Fast Real-Time PCR System (Applied Biosystems, USA), applying the thermal cycling protocol described by Nielsen et al. (2013).

### 2.5 Statistical analysis

The qPCR values are expressed as nanograms (ng) of fungal DNA per microgram (μg) of plant DNA. Results are presented as means, and the standard deviation of the values was calculated. A three-way ANOVA (Type III sums of squares) was performed to evaluate the main and interaction effects of the sowing date, cultivar, and seed treatment fungicide. Statistical analyses were conducted using JASP (version 0.19.3; JASP Team, Amsterdam, The Netherlands). Statistical significance was set at p <0.05. The results are presented as mean ± standard deviation (SD). Figures and tables were generated using Microsoft Excel (Microsoft Corp., Redmond, WA, USA).

## 3 Results

Root rot was observed in winter wheat during the entire research period. Despite consistent disease pressure, most of the applied seed treatment fungicides were effective against root rot pathogens (Sabeckis et al., 2025). Based on these findings, a molecular study was conducted. Field samples were analyzed using quantitative PCR (qPCR) analysis. Data confirmed the presence of *M. nivale* and *M. majus* in all the analyzed winter wheat cultivars. The quantity of fungal DNA varied depending on the cultivar, sowing time, and year of sampling. The highest concentrations of *M. nivale* and *M*. *majus* DNA were recorded in 2023, whereas the lowest were detected in 2024. These fluctuations likely reflect the influence of meteorological conditions that are favorable for *Microdochium* spp. development.

### 3.1 *M. nivale* DNA quantitative assay

Quantitative PCR analysis confirmed the presence of *M. nivale* in all tested winter wheat cultivars, although there was substantial variation in pathogen DNA quantity in different years (**Table 3**). The highest values of *M. nivale* DNA in all tested cultivars were detected in 2023, coinciding with the presence of snow mold in the trial and/or prolonged snow cover (Sabeckis et al., 2025), whereas lower levels occurred in 2024, reflecting less favorable conditions for pathogen development. For example, in the cultivar “Ada” (optimal sowing, untreated), *M. nivale* DNA reached 723.77 ng μg^−1^ in 2023 but declined to 7.99 ng μg^−1^ in 2024. Similarly, “Skagen” (optimal sowing, untreated) exhibited 241.35 in 2023 and maintained comparatively high levels in 2024 (70.04 ng μg^−1^), despite the overall reduction in disease pressure.

**Table 3:** Mean ( ± SD) quantity of *Microdochium nivale* DNA (ng pathogen DNA per μg plant DNA) in winter wheat cultivars sown at optimal and late dates under different seed treatment fungicide applications, 2022–2024.

Seed treatment fungicide	Culivars							
	Optimal				Late			
	Ada	KWS Emil	Skagen	Patras	Ada	KWS Emil	Skagen	Patras
2022								
Untreated	191.93 ± 170.13	5.78 ± 5.15	209.25 ± 150.63	17.35 ± 25.95	26.66 ± 37.22	0.57 ± 1.15	72.95 ± 72.93	10.34 ± 19.97
Bariton Super	13.57 ± 22.11	0.49 ± 0.98	5.76 ± 5.76	2.17 ± 0.91	0.26 ± 0.33	0.00 ± 0.00	15.98 ± 16.73	0.00 ± 0.00
Maxim 025 FS	19.69 ± 23.11	0.00 ± 0.00	10.07 ± 14.26	7.03 ± 12.32	0.23 ± 0.45	0.09 ± 0.18	0.59 ± 0.82	0.34 ± 0.27
Kinto Plus	6.24 ± 58.56	0.00 ± 0.00	19.26 ± 29.17	1.38 ± 1.67	0.40 ± 0.80	1.07 ± 1.05	0.29 ± 0.59	0.43 ± 0.85
Vibrance Star	65.27 ± 80.93	0.35 ± 0.70	8.99 ± 10.71	6.09 ± 6.33	0.00 ± 0.00	0.00 ± 0.00	13.22 ± 26.20	0.03 ± 0.06
2023								
Untreated	723.77 ± 121.34	241.36 ± 81.73	241.35 ± 87.98	278.55 ± 88.95	213.04 ± 245.99	229.31 ± 88.92	138.71 ± 76.20	134.99 ± 63.15
Bariton Super	153.39 ± 77.58	113.55 ± 30.68	90.31± 59.03	86.80 ± 95.96	6.92 ± 7.36	20.90 ± 15.70	48.44 ± 32.96	52.25 ± 60.69
Maxim 025 FS	74.91 ± 32.78	31.36 ± 9.06	81.11 ± 23.54	85.23 ± 28.82	14.03 ± 21.75	5.83 ± 5.52	30.51 ± 13.89	52.26 ± 34.27
Kinto Plus	77.52 ± 55.03	15.62 ± 11.35	90.60 ± 45.46	15.44 ± 6.40	2.96 ± 3.02	21.10 ± 20.21	30.56 ± 10.49	80.59 ± 81.97
Vibrance Star	97.45 ± 68.74	67.88 ± 20.05	138.61 ± 74.79	45.81 ± 36.21	4.66 ± 5.64	29.44 ± 22.47	40.35 ± 14.70	63.29 ± 47.70
2024								
Untreated	7.99 ± 12.16	29.93 ± 41.09	70.04 ± 54.08	1.80 ± 1.44	0.69 ± 0.81	4.11 ± 8.22	5.78 ± 5.71	1.66 ± 2.17
Bariton Super	3.09 ± 1.82	1.27 ± 0.36	11.00 ± 7.62	5.38 ± 8.23	0.71 ± 1.16	1.67 ± 2.13	24.23 ± 18.50	1.91 ± 2.38
Maxim 025 FS	71.27 ± 52.9	21.91 ± 34.16	23.54 ± 15.45	1.53 ± 1.99	0.00 ± 0.00	2.09 ± 2.91	23.01 ± 19.05	0.82 ± 0.95
Kinto Plus	12.19 ± 10.84	1.79 ± 1.26	55.48 ± 38.84	2.80 ± 5.12	0.30 ± 0.59	0.00 ± 0.00	0.55 ± 1.10	2.57 ± 2.28
Vibrance Star	2.74 ± 2.39	14.54 ± 18.46	6.83 ± 4.78	1.46 ± 1.42	0.35 ± 0.41	1.84 ± 0.78	2.51 ± 3.47	0.78 ± 1.56

The data suggested that sowing time influenced infection levels, with later sowing generally resulting in reduced *M. nivale* DNA compared to optimal sowing. In most cases, sowing time was most evident in low to moderate pressure years (2022 and 2024), whereas in spring 2023, under more favorable environmental conditions for the pathogen, higher DNA quantities were detected regardless of sowing time, thereby reducing the impact of this factor. Seed treatment with fungicides typically reduced pathogen DNA levels compared with those in untreated controls. Notably, Maxim 025 FS (fludioxonil) and Bariton Super (protioconazole + tebuconazole + fludioxonil) often provided substantial reductions in DNA of *M. nivale* in winter wheat samples under high disease pressure. Kinto Plus (fluxapyroxad + triticonazole + fludioxonil), as well as Vibrance Star (sedaxane + fludioxonil + triticonazole) also demonstrated pathogen suppression, though with variable results throughout the research years.

However, in the context of effective reduction of pathogen DNA, the efficacy of individual seed treatment fungicides varied, and no single treatment consistently outperformed the others. The results also indicated differences between cultivars in certain years, with more sensitive cultivars “Ada” and “Skagen” often exhibiting higher infection levels than “KWS Emil” and “Patras,” although cultivar-related distinctions diminished during epidemic conditions when disease pressure was universally high.

In general, *M. nivale* DNA was present in different amounts in all wheat cultivars, with the highest DNA amounts in a year with snow mold and/or prolonged snow cover. In fact, later sowing times led to reduced *M. nivale* DNA compared to optimal sowing times. Fungicide seed treatments reduced the DNA amount of the pathogen, but the efficacy was inconsistent between years and cultivars.

### 3.2 *M. majus* DNA quantitative assay

The data from the quantitative PCR analysis for *M. majus* are presented in **Table 4**. Analysis showed the presence of the pathogen in winter wheat across all three years, with even more pronounced variation between seasons and treatments that are observed for *M. nivale*. The highest DNA quantities were generally observed in 2022, with exceptionally high values in some cultivar and seed treatment combinations, particularly in untreated cv. “Ada” (optimal sowing, 1,027.21 ng μg^−1^) and cv. “KWS Emil” (574.30 ng μg^−1^). In contrast, the pathogen DNA levels were much lower in 2023. Similar to the *M. nivale* data, the lowest quantities occurred in 2024, when DNA quantities in most treatments and cultivars were near zero, indicating unfavorable conditions for *M. majus* development.

**Table 4:** Mean ( ± SD) quantity of *Microdochium majus* DNA (ng pathogen DNA per μg plant DNA) in winter wheat cultivars sown at optimal and late dates under different seed treatment fungicide applications, 2022–2024.

Seed treatment fungicide	Cultivars							
	Optimal				Late			
	Ada	KWS Emil	Skagen	Patras	Ada	KWS Emil	Skagen	Patras
2022								
Untreated	1,027.21 ± 722.13	574.3 ± 169.54	6.55 ± 12.94	0.00 ± 0.00	0.66 ± 1.29	0.03 ± 0.06	67.59 ± 82.5	0.07 ± 0.08
Bariton Super	24.62 ± 49.23	0.00 ± 0.00	14.20 ± 22.56	0.36 ± 0.27	0.00 ± 0.00	29.44 ± 58.13	17.70 ± 27.83	0.04 ± 0.08
Maxim 025 FS	251.27 ± 296.61	0.00 ± 0.00	28.68 ± 36.82	2.67 ± 5.33	0.00 ± 0.00	0.09 ± 0.18	21.09 ± 14.93	0.13 ± 0.25
Kinto Plus	215.05 ± 174.25	0.00 ± 0.00	30.10 ± 60.2	0.00 ± 0.00	0.09 ± 0.17	0.00 ± 0.00	2.96 ± 2.53	0.26 ± 0.18
Vibrance Star	83.85 ± 151.08	32.37 ± 38.36	16.71 ± 11.09	1.41 ± 2.82	0.07 ± 0.08	0.00 ± 0.00	9.89 ± 19.77	0.00 ± 0.00
2023								
Untreated	277.62 ± 90.19	244.92 ± 141.57	34.46 ± 18.03	99.59 ± 70.02	276.76 ± 180.49	120.49 ± 57.57	11.00 ± 10.26	29.44 ± 34.16
Bariton Super	276.48 ± 76.89	278.94 ± 116.09	70.65 ± 79.55	13.22 ± 26.43	163.49 ± 77.54	189.54 ± 83.01	15.50 ± 22.16	49.03 ± 35.88
Maxim 025 FS	190.44 ± 73.95	342.03 ± 111.23	43.48 ± 13.72	85.23 ± 47.13	272.85 ± 192.59	238.49 ± 82.12	6.43 ± 4.29	47.82 ± 44.76
Kinto Plus	69.75 ± 64.33	108.62 ± 34.23	36.58 ± 16.86	7.50 ± 14.99	106.71 ± 98.47	110.28 ± 86.62	6.15 ± 8.2	7.66 ± 5.99
Vibrance Star	40.12 ± 26.91	206.38 ± 208.70	19.71 ± 18.74	17.67 ± 35.34	42.03 ± 70.07	63.54 ± 28.81	7.55 ± 7.01	14.00 ± 21.72
2024								
Untreated	0.10 ± 0.20	0.83 ± 1.66	1.19 ± 2.38	6.85 ± 13.69	0.00 ± 0.00	0.00 ± 0.00	0.00 ± 0.00	0.00 ± 0.00
Bariton Super	9.03 ± 12.18	0.21 ± 0.42	0.30 ± 0.61	0.28 ± 0.56	0.00 ± 0.00	0.95 ± 1.89	0.20 ± 0.41	0.00 ± 0.00
Maxim 025 FS	7.97 ± 10.19	7.22 ± 14.43	0.17 ± 0.33	0.00 ± 0.00	0.00 ± 0.00	0.00 ± 0.00	0.00 ± 0.00	0.00 ± 0.00
Kinto Plus	1.69 ± 2.23	4.01 ± 7.33	0.27 ± 0.54	0.00 ± 0.00	0.00 ± 0.00	0.00 ± 0.00	0.00 ± 0.00	0.00 ± 0.00
Vibrance Star	0.25 ± 0.50	9.46 ± 18.91	12.43 ± 11.59	3.63 ± 7.27	0.00 ± 0.00	0.00 ± 0.00	0.00 ± 0.00	0.00 ± 0.00

The time of sowing also affected the number of DNA and, therefore, the infection patterns in certain years. In 2022, later-sown winter wheat contained greatly reduced DNA quantities in most cultivars; for example, the values in cv. “Ada” samples dropped from 1027.21 (optimal) to 0.66 (late), and cv. “KWS Emil” from 574.30 to 0.03 ng μg^−1^. However, in 2023, the effect of sowing time was less consistent, and in some cases, late sowing still resulted in high levels of pathogen DNA, as seen in cv. “Ada,” where late-sown wheat samples contained values (276.76 ng μg^−1^) comparable to those of optimally sown wheat (277.62 ng μg^−1^).

*M. majus* DNA levels were generally lower in treated seed winter wheat compared to untreated controls, particularly in years when quantities were exceptionally high. In 2022, at the optimal sowing time, winter wheat cv. “Ada” treated with prothioconazole + tebuconazole + fludioxonil (Bariton Super) contained 24.62 ng μg^−1^ compared to 1027.21 ng μg^−1^ in the untreated control, while cv. “KWS Emil” samples did not contain pathogen DNA compared to 574.30 ng μg^−1^ in the untreated control with the same fungicide. Treatments with Maxim 025 FS (fludioxonil), Kinto Plus (fluxapyroxad + triticonazole + fludioxonil), and Vibrance Star (sedaxane + fludioxonil + triticonazole) also showed strong reductions in some cases, although their relative efficacy varied in 2023. For example, seed treatment with Maxim 025 FS in cv. “KWS Emil” winter wheat sowed optimal contained unexpectedly high values of pathogen DNA—342.03 ng μg^−1^. In 2024, the fungicide impact was less revealing because of the very low overall infection. For cultivar comparison, differences were more pronounced in 2022, with “Ada” and “KWS Emil” showing much higher DNA quantities than “Skagen” and “Patras,” especially under optimal sowing without treatment. In 2023, the results were less distinctive, with DNA quantities resulting in moderate levels, although winter wheat samples of cultivars “Ada” and “KWS Emil” tended to rank higher than the others. By 2024, cultivar differences were minimal because of uniformly low infection levels.

Overall, the results indicate that *M. majus* pressure was greatest in 2022, less intense in 2023, and negligible in 2024, with clear indications of cultivar and sowing time effects apparent only under high to moderate disease pressure with high general DNA quantity. Fungicide treatments were generally effective in reducing values, although their relative performance varied between years and cultivars.

### 3.3 Interaction of different factors on *M. nivale* and *M. majus* DNA Quantity

An interaction analysis was performed to evaluate whether the effects of sowing time, cultivar, and seed treatment fungicides on *Microdochium* DNA levels were influenced by each other, providing insight into how these factors jointly affect pathogen development. Data for *M. nivale* presented in **Table 5** showed clear two- and three-factor interactions between sowing time, cultivar, and seed treatment fungicides, which were the most evident in 2023, reflecting complex factor relationships under high disease pressure. Whereas *M. majus* interactions (**Table 6**) interaction effects were strongest in 2022, indicating that factor combinations had the greatest influence during years of exceptionally high pathogen DNA levels.

**Table 5:** Analysis of variance for interactions between sowing time, cultivar, and seed treatment fungicides (STF) on *M. nivale* DNA quantity in winter wheat, 2022–2024.

Interaction	2022				2023				2024			
	DF	Mean square	F value	P value	DF	Mean square	F value	P value	DF	Mean square	F value	P value
Sowing ✻ Cultivar	4	6545.2	3.2	**0.015**	4	40916.2	9.9	**<0.001**	4	1605.1	3.4	**0.011**
Sowing ✻ STF	4	13860.6	6.8	**<0.001**	4	25917.6	6.3	**<0.001**	4	759.1	1.6	0.177
Cultivar ✻ STF	16	4907.9	2.4	**0.003**	16	36846.2	8.9	**<0.001**	16	705.7	1.5	**0.110**
Sowing ✻ Cultivar ✻ STF	16	2306.9	1.1	0.336	16	16222.8	3.9	**<0.001**	16	982.1	2.1	**0.012**

Numbers in bold indicate statistically significant differences.

**Table 6:** Analysis of variance for interactions between sowing time, cultivar, and seed treatment fungicides (STF) on *M. majus* DNA quantity in winter wheat, 2022–2024.

Interaction	2022				2023				2024			
	DF	Mean square	F value	P value	DF	Mean square	F value	P value	DF	Mean square	F value	P value
Sowing ✻ Cultivar	4	199455.4	13.9	**<0.001**	4	12936.3	2.4	**0.049**	4	359.0	6.9	**<0.001**
Sowing ✻ STF	4	159378.7	11.1	**<0.001**	4	5994.8	1.1	0.343	4	329.9	6.4	**<0.001**
Cultivar ✻ STF	16	69874.2	4.9	**<0.001**	16	17402.3	3.3	**<0.001**	16	192.1	3.7	**<0.001**
Sowing ✻ Cultivar ✻ STF	16	76204.1	5.3	**<0.001**	16	5115.3	1.0	0.495	16	192.8	3.7	**<0.001**

Numbers in bold indicate statistically significant differences.

Data for *M. nivale* showed that interaction patterns varied across years. The sowing time and cultivar interaction was significant in all three years, with the strongest in 2023 (*F* = 9.9, *sig.* <0.001), indicating that cultivar responses to sowing time were most evident under high disease pressure. The sowing time × seed treatment fungicide interaction was significant in 2022 and 2023 (*sig.* <0.001), suggesting that fungicide performance depended on the sowing date in these years, but not in 2024. Cultivar × fungicide interactions were significant in 2022 (*p* = 0.003) and especially in 2023 (*F* = 8.9, *sig <*0.001), showing strong cultivar-specific differences in fungicide responses under epidemic conditions. The three-way interaction was significant in 2023 (*p <*0.001) and 2024 (*p* = 0.012), indicating complex, environment-dependent factor combinations.

Conversely, for *M. majus*, the sowing time × cultivar interaction was significant only in 2022 (F = 15.6, sig. <0.001), indicating that cultivar adaptation to sowing time was most critical in years with extremely high pathogen presence. Furthermore, the interactions tended to be stronger in 2022 than in subsequent years. The sowing time × cultivar interaction was highly significant in 2022 (*F* = 13.9, *p <*0.001) and 2024 (*F* = 6.9, *p <*0.001), but was weaker in 2023 (*p* = 0.049). The sowing time × fungicide interaction was significant in 2022 and 2024 (*p <*0.001 for both), but not in 2023, suggesting a reduced role of sowing date in moderating fungicide effects during that year. Cultivar × fungicide interactions were significant in all years, with the strongest effect in 2022 (*F* = 4.9, *p <*0.001), indicating consistent cultivar-specific differences in the fungicide response. The three-way interaction was significant in 2022 and 2024 (*p <*0.001 for both) but absent in 2023, suggesting that under certain environmental conditions, the combined influence of sowing time, cultivar, and fungicide was reduced.

Overall, *M. majus* showed more pronounced and consistent interaction effects in high-pressure years like 2022, whereas *M. nivale* exhibited complex multi-factor interactions under epidemic conditions in 2023.

## 4 Discussion

The results of this three-year field study provide valuable insights into the complex dynamics of *M. nivale* and *M. majus* in winter wheat, highlighting the influence of environmental conditions, particularly prolonged snow cover, sowing time, seed treatment fungicides, and cultivar susceptibility to pathogen populations.

Our study revealed notable fluctuations in *Microdochium* DNA levels, with the highest concentrations of *M. nivale* and *M. majus* DNA recorded in 2023 and 2022, respectively, and the lowest levels in 2024. Specifically, the high DNA quantities of *M. nivale* in 2023 coincided with prolonged snow cover (up to 77 consecutive days) and temperatures fluctuating around 0°C in June (Sabeckis et al., 2025). This strongly aligns with the existing literature that emphasizes *Microdochium nivale* and *M. majus* as psychrotolerant fungi that thrive under prolonged snow cover (Gorshkov et al., 2020), which provides insulation, darkness, and humidity, thereby creating highly favorable conditions for snow mold development (Gorshkov et al., 2020; Tsers et al., 2023).

Experimental and field studies have demonstrated that *M. nivale* and *M. majus* differ in their host associations and competitive behaviors. In controlled inoculation experiments, *M. majus* isolates exhibited a weak but consistent selective advantage in wheat and oat seedlings, whereas *M. nivale* showed a strong competitive advantage in rye (Simpson et al., 2000). These observations are supported by large-scale field surveys of historical samples in Denmark, where *M. majus* was more frequently detected in wheat, barley, oats, and triticale, whereas *M. nivale* predominated in rye, suggesting that *M. majus* has been the dominant pathogen in cereals for the last 50 years (Nielsen et al., 2013). Conversely, a comprehensive study in Northeast Europe, which genetically characterized 46 *Microdochium* isolates from cereal (grains and plant tissues), found that 59% corresponded to *M. nivale*, while only 28% matched *M. majus* (Gagkaeva et al., 2020). This difference between regional studies may be linked to climatic differences, particularly snow cover: the continental interior of northeastern Europe, the presence of consistent and prolonged snow cover could favor the proliferation of *M. nivale*, whereas in the North Sea–Baltic maritime climate, snow cover is less constant and sporadic, likely creating conditions more favorable for *M. majus* growth. Our results align with this reasoning, as high *M. nivale* DNA levels were detected in 2023 under prolonged snow cover, whereas *M. majus* was more prominent in 2022, a season characterized by different meteorological conditions. Notably, DNA quantities in 2021 were relatively high despite moderate visual root rot pressure in field experiments designed to evaluate disease damage (Sabeckis et al., 2025), suggesting that pathogen DNA levels in stem base diseases do not always correspond directly with symptom expression, a pattern also observed for *Oculimacula* spp. in winter rye (Ramanauskienė et al., 2014). A study on *Microdochium* isolates showed that *M. nivale* and *M. majus* were the most pathogenic to wheat compared to other species (Ünal, 2024). Other researchers have found that *Microdochium nivale* might be more aggressive as a causal agent of root rot and also has a significantly stronger reducing effect on plant stem and root length compared to *M. majus* (Ren et al., 2015). Furthermore, these two species exhibit different capacities to cause damage depending on the temperature (Imathiu et al., 2010).

Our findings indicate distinct differences in cultivar susceptibility, with “Ada” consistently exhibiting higher levels of *M*. *nivale* DNA than other cultivars, suggesting its increased susceptibility. Similarly, for *M*. *majus* in 2022, “Ada” and “KWS Emil” showed significantly higher DNA quantities than “Skagen” and “Patras,” particularly regarding optimal sowing without treatment. Specific cultivar resistance mechanisms can interact with variability in pathogenicity among *Microdochium* populations, as demonstrated in other studies (Jewell and Hsiang, 2013; Abdelhalim et al., 2020; Gagkaeva et al., 2020). The impact of environmental conditions on winter survival among wheat lines can sometimes be more significant than inoculation itself, making it difficult to draw definitive conclusions on specific snow mold resistance challenges without considering environmental interactions (Ergon et al., 2003). Moreover, the importance of environmental context in winter is further highlighted by Yarosh et al. (2024), who addressed that snow cover and ecological plasticity strongly influenced cultivar resistance.

Seed treatment fungicides generally reduced *Microdochium* DNA quantity, supporting their significant role in disease control, although their effectiveness varied considerably. For instance, Bariton Super notably reduced *M. majus* in “Ada” at optimal sowing time from 1,027.21 to 24.62 ng μg^−1^ in 2022, while in Maxim 025 FS and Kinto Plus treated seed samples, the quantity averaged 251.27 and 215.05 ng μg^−1^, respectively. Fungicide treatments are recognized for improving plant vigor and enhancing winter survival in wheat and have been shown to be effective against seed- and soil-borne diseases such as smuts, kernel bunt, and root rots, with various active ingredients demonstrating efficacy in multiple studies (Bugingo et al., 2025). In some instances, however, *M. majus* DNA quantities were unexpectedly high (e.g., Maxim 025 FS in “KWS Emil” optimal sowing time in 2023), suggesting that fungicide performance can vary under different environmental conditions and disease pressure. Fludioxonil-based and SDHI (fluxapyroxad or sedaxane in Kinto Plus and Vibrance Star, respectively) containing seed treatments provided the most consistent suppression, whereas triazole-dominant mixtures showed effective yet more variable performance. This is in line with the results obtained by other researchers (Müllenborn et al., 2008; Brown et al., 2021).

The influence of sowing time on *Microdochium* infection levels was most evident for *M. nivale*, as in 2022, later sowing times significantly reduced DNA quantities in most tested cultivars. In addition, DNA quantities were lower in later-sown winter wheat in most years for *M. nivale*. Results regarding root rot suggested that later sowing reduced disease levels in years with low to moderate pressure, although this effect was less pronounced under high disease pressure, such as in 2023 (Sabeckis et al., 2025). Comparable results were obtained in Latvian field trials, suggesting that later sowing consistently lowers snow mold incidence in winter wheat, despite earlier sown plants accumulating more carbohydrates linked with cold tolerance (Ruza et al., 2011). Similarly, a study in Finland reported that postponing sowing reduced pink snow mold, leaf rust, and winter damage in winter rye, reinforcing the protective role of delayed sowing in northern climates (Serenius et al., 2005).

## 5 Conclusions

This study provides specific insights into the epidemiology of *Microdochium nivale* and *M. majus* in winter wheat using qPCR-based quantification of samples from 3-year field trials. Both pathogens were detected across all cultivars, but their prevalence varied with environmental conditions: *M. nivale* dominated under prolonged snow cover, whereas *M. majus* prevailed when snow cover was less persistent. Sowing time was more influential for *M. nivale* infection in years with moderate disease pressure, with later sowing generally reducing the pathogen DNA quantity. Although this effect was slightly less visible under conditions of higher pathogen pressure. Seed treatment fungicides, particularly those containing fludioxonil and SDHI compounds (fluxapyroxad or sedaxane), consistently suppressed pathogen DNA levels, although no treatment provided complete or uniform control across the years. Cultivar-related differences were also evident, with ‘Ada’ and ‘KWS Emil’ often exhibiting higher infection levels, suggesting that the genetic background interacts with pathogen species and environmental conditions to shape pathogen prevalence. By evaluating the influences and interactions of multiple environmental, agronomic, and genetic factors, this study contributes to advancing the sustainable management of *Microdochium-*related diseases and safeguarding winter wheat yields under changing climatic conditions.

## Data Availability

The original contributions presented in the study are included in the article/supplementary material. Further inquiries can be directed to the corresponding author.
